# Mechanical Circulatory Support in Cardiogenic Shock: A Narrative Review

**DOI:** 10.7759/cureus.69379

**Published:** 2024-09-13

**Authors:** Dhruv Shah, Jayshree Sen

**Affiliations:** 1 Anaesthesiology, Jawaharlal Nehru Medical College, Datta Meghe Institute of Higher Education and Research, Wardha, IND

**Keywords:** cardiogenic shock, extracorporeal membrane oxygenation (ecmo), hemodynamic stabilization, intra-aortic balloon pump, mechanical circulatory support, ventricular assist devices (vads)

## Abstract

Cardiogenic shock (CS) remains a critical condition with high mortality rates, often arising from acute myocardial infarction and advanced heart failure. Despite advancements in medical therapy, traditional interventions frequently fall short of reversing the profound hemodynamic instability seen in CS. Mechanical circulatory support (MCS) devices have emerged as crucial therapeutic options, offering temporary stabilization and bridging to recovery, heart transplantation, or long-term ventricular assist device implantation. This narrative review explores the current landscape of MCS in CS, highlighting the various types of support devices, including intra-aortic balloon pumps (IABP), veno-arterial extracorporeal membrane oxygenation (VA-ECMO), and percutaneous ventricular assist devices (pVADs) such as Impella and TandemHeart. We examine their mechanisms of action, clinical indications, and outcomes alongside the challenges and complications associated with their use. Additionally, we discuss the evolving guidelines and the role of MCS in contemporary CS management, emphasizing the need for timely intervention and a multidisciplinary approach. The review underscores the importance of individualized patient selection and device choice to optimize outcomes in this critically ill population.

## Introduction and background

Cardiogenic shock (CS) is a critical condition characterized by the inadequate pumping function of the heart, resulting in severe systemic hypoperfusion and tissue hypoxia. It is commonly defined by a sustained reduction in cardiac output, leading to hypotension, elevated systemic vascular resistance, and diminished end-organ perfusion [1]. In a clinical sense, CS has an elevated mortality rate and represents a serious problem in intensive care units. Management is crucial because, in addition to hemodynamic stabilization, it also involves addressing the underlying cause of heart failure [2]. In the management of CS, mechanical circulatory support (MCS) is paramount, as it offers either temporary or long-term support to the ailing heart, depending on the pathophysiology of the initial insult. MCS devices such as intra-aortic balloon pumps (IABP), ventricular assist devices (VADs), and extracorporeal membrane oxygenation (ECMO) work by artificially improving the patient's hemodynamics and enhancing organ perfusion hemodynamic stability, enhancing organ perfusion and serving as a bridge for patients to either recovery or definitive treatment [3]. The advent of advanced MCS technologies has significantly impacted the outcomes in patients with CS, offering new hope for those with severe cardiac dysfunction and reduced survival chances [4].

This narrative review aims to provide a comprehensive overview of the current state of MCS in the management of CS. There are many different kinds of MCS technologies in use today, and this review will properly go through their modes of operation, different clinical applications, and their effects. It would also touch on recent advances in MCS, such as innovations and upcoming trends, to give a better understanding of how to go about managing CS today. The aim would be to demonstrate the role of MCS in enhancing patient outcomes, thus enabling doctors to make informed decisions during CS, supported by the present information available and expert opinions [5].

## Review

Search methodology

The search methodology for the narrative review on "Mechanical Circulatory Support in Cardiogenic Shock" involved a comprehensive literature search across multiple electronic databases, including PubMed, Scopus, and Web of Science. The search was conducted using relevant keywords and medical subject headings (MeSH) such as "cardiogenic shock," "mechanical circulatory support," "intra-aortic balloon pump," "ventricular assist devices," "extracorporeal membrane oxygenation," and "temporary mechanical support." Articles published in English from the past two decades were prioritized to ensure the inclusion of the most current and relevant studies. Reference lists of selected articles were also screened to identify additional pertinent studies. Both clinical trials and observational studies, as well as expert consensus guidelines and systematic reviews, were considered to provide a comprehensive overview of the current state of knowledge in the field. The focus was on studies that explored the efficacy, safety, and outcomes of different MCS devices in the management of CS.

Pathophysiology of CS

Underlying Mechanisms

CS is a severe condition occurring when the heart fails to pump enough blood to satisfy the metabolic needs of its body, thus resulting in reduced oxygen supply to end organs and peripheral tissues, causing anaerobic metabolism and cell anaerobic metabolism of tissues. Acute cardiac dysfunction is the leading cause involved, and it can be due to many reasons, such as myocardial infarction (MI), severe heart failure, or acute myocarditis. In MI, ischemia and necrosis subsequently reduce cardiac contractility and hinder the ejection force of blood, decreasing output from the heart [6]. Additionally, increased left ventricular end-diastolic pressure due to impaired contractility leads to pulmonary vascular congestion and subsequent volume overload, further exacerbating the shock state [7].

Impact on Organ Systems

The impaired cardiac output in CS leads to reduced systemic perfusion pressure, which affects multiple organ systems; decreased perfusion pressure results in diminished blood flow to vital organs, such as the kidneys, liver, and brain, leading to multi-organ dysfunction. In the kidneys, reduced perfusion can cause acute renal failure, characterized by oliguria or anuria and elevated serum creatinine levels [8]. The liver may experience ischemic damage, manifesting as elevated, which may result in transaminitis and jaundice. Neurologically, decreased cerebral perfusion can result in confusion, altered consciousness, or even coma [9]. The combined effects of these systemic impacts exacerbate the overall clinical picture of CS, making timely and effective intervention critical.

Types of MCS devices

IABP

The IABP is a widely used MCS device designed to improve myocardial oxygen delivery and reduce cardiac workload within the descending aorta. It consists of a balloon catheter that inflates and deflates concurrently to the rhythm of the heart. Inflation happens during diastole, which raises the pressure in the aorta and improves blood supply to coronary arteries. Deflation is a crucial element that is frequently overlooked by many physicians I collaborate with. The rapid deflation of the IABP provides significant negative pressure and thus increases forward flow. This is why IABPs are useful for the treatment of mitral regurgitation [10]. This device is particularly beneficial in managing CS by providing temporary support and improving hemodynamic stability, although it is generally considered a bridge to more definitive therapies or recovery [11]. For instance, studies have shown that IABP can effectively stabilize patients awaiting cardiac transplantation or those recovering from severe MI [10].

VADs

In the field of MCS, VADs refer to tools developed for managing heart failure by providing additional assistance or complete replacement of the pumping action of the heart. VADs may take different shapes and sizes, such as left ventricular assist devices (LVADs), right ventricular assist devices (RVADs), and biventricular assist devices (BiVADs), depending on what specific portion should be supported [12]. LVADs, the most common type, are implanted to support the left ventricle by pumping blood from the left ventricle into the aorta, thereby improving cardiac output and perfusion [13]. There are different clinical applications for VAD in bridge-to-transplant patients as well as for patients who are not eligible for transplantation with the same device serving as a permanent therapy. Clinical trials have shown that VADs can improve both survival and quality of life to a considerable extent in patients with end-stage heart failure [14].

ECMO

ECMO is a form of MCS used in cases of severe cardiopulmonary failure. There are two primary types of ECMO: veno-venous (VV) and veno-arterial (VA), each with specific indications based on the nature of the patient's cardiopulmonary dysfunction [15-17].

VV-ECMO: VV-ECMO is used when the heart retains adequate function, but the lungs are unable to meet the body's oxygenation demands. This type of ECMO is indicated in cases such as severe acute respiratory distress syndrome (ARDS), pneumonia, or other forms of respiratory failure. It provides oxygenation and carbon dioxide removal by diverting venous blood from the body, oxygenating it externally, and returning it to the venous system. Since VV ECMO does not directly support cardiac function, it is reserved for patients with isolated lung failure [15].

VA-ECMO: VA-ECMO, on the other hand, is used when both cardiac and respiratory support are needed. It is indicated in cases of CS, cardiac arrest, or severe heart failure, where the heart is unable to pump effectively, and the lungs may also be compromised. VA-ECMO provides both oxygenation and circulatory support, with oxygenated blood being returned to the arterial system, thus bypassing both the heart and lungs. This enables adequate body perfusion, maintaining oxygen supply and blood pressure [15].

Other Emerging Devices

Several emerging MCS devices are currently under development or in clinical trials, aiming to address the limitations of existing technologies. These include next-generation VADs with smaller, more durable components and new types of percutaneous support devices that can be implanted with minimally invasive techniques [18]. Innovations such as fully implantable VADs and bioengineered heart pumps are also on the horizon, offering potential advantages in terms of patient comfort, reduced risk of infection, and improved overall outcomes [19]. These emerging technologies are anticipated to provide more personalized and effective treatment options for patients with severe cardiac conditions, enhancing the field of MCS significantly [20].

Clinical applications and evidence

Indications for MCS

MCS is primarily indicated in cases of CS where conventional treatments fail to restore adequate hemodynamic stability. This includes patients with severe left ventricular dysfunction who exhibit persistent low cardiac output despite optimal pharmacological and non-pharmacological interventions. According to a study by Werdan et al. [4], MCS is recommended when patients have a high risk of mortality and persistent symptoms despite maximal medical therapy. Furthermore, the American Heart Association (AHA) guidelines suggest that MCS should be considered in cases of acute MI complicated by CS where there is evidence of significant myocardial damage and hemodynamic compromise [21]. In addition, MCS is indicated for patients awaiting heart transplantation, providing critical support to sustain life and improve end-organ perfusion while they wait for a donor heart [22].

Outcomes and Effectiveness

The effectiveness of MCS in treating CS has been evaluated through various studies, demonstrating improvements in short-term survival and organ function. A review published in 2014 found that patients receiving MCS showed significant improvements in hemodynamic parameters, such as cardiac output and blood pressure, compared to similar patients receiving only medical management [4]. Additionally, the use of MCS has been associated with a reduction in mortality rates among patients with severe CS. For example, a study by Khan et al. (2014) [23] reported a 30-day mortality rate of 32% in patients with MCS compared to 45% in those receiving conventional therapy alone. However, the long-term benefits of MCS remain debated, with some studies highlighting issues such as device-related complications and the need for further interventions [24].

Comparative Analysis

Comparative studies on MCS devices, such as IABP and LVAD, reveal differences in efficacy and clinical outcomes. A review by Vihari et al. (2023) compared IABP and LVAD in patients with CS and found that LVAD was associated with higher rates of short-term survival and better hemodynamic support than IABP [25]. Conversely, IABP is less invasive and carries a lower risk of device-related complications, making it a viable option for less severe cases of CS [26]. Another comparative study highlighted that while LVAD provides superior circulatory support and improved end-organ perfusion, it is associated with higher costs and more complex management. The choice between MCS devices depends on patient-specific factors, including the severity of shock, anticipated duration of support, and overall patient condition [27].

Challenges and complications

Short-Term Complications

MCS systems, such as IABP and VAD, are highly beneficial to patients suffering from CS; however, their application is characterized by several immediate complications. Some of the frequent problems are bleeding infections and device failures, among others. For instance, the anticoagulation required for these devices can lead to bleeding complications, such as gastrointestinal bleeding or intracranial bleeding, as discussed in the review of VAD-related bleeding risks [28]. Infections, especially at the device insertion site or involving the pump's components, are common and can result in sepsis [29]. Device malfunction, including issues such as pump thrombosis or mechanical failure, poses a significant risk and can result in device replacement or patient deterioration [30].

Long-Term Considerations

Long-term considerations for patients on MCS systems involve managing chronic complications and optimizing device performance. One major issue is the risk of pump thrombosis, which can result in device malfunction and necessitate anticoagulation therapy, leading to an increased risk of bleeding [31]. Patients may also experience persistent infections, such as driveline infections, which require long-term antibiotic therapy and, in some cases, surgical intervention [23]. Additionally, there is the challenge of managing the psychological and lifestyle impacts of living with a mechanical support device, which can affect the quality of life and adherence to treatment [32].

Challenges in Device Management

Several challenges arise in managing MCS devices, including the necessity for constant observation and modifying treatment protocols depending on how the patient responds. Therefore, the management of these devices has to be done with diligence, such as looking at device parameters like flow rates and pressure settings, in order to avert potential complications that may arise in motors, such as pump thrombosis or insufficient support [33]. Additionally, the integration of these devices with other therapeutic interventions, such as pharmacotherapy and lifestyle modifications, can be complex and requires a multidisciplinary approach [34]. Technological advancements in device design and automation have improved management but also introduced new challenges related to device compatibility and software updates [35].

Future directions and innovations

For MCS to be successful in the future, it will need to focus on technological advancements, personalized care, and a better long-term prognosis. For instance, there are new smaller and more versatile VADs that have improved biocompatibility and durability, enabling a wider range of patients to benefit from MCS. In addition, new percutaneous and implantable MCS systems intend to reduce invasiveness and quicken recovery timeframes while at the same time leveraging artificial intelligence and machine learning to achieve optimum device settings and real-time predictions of patient responses. Additionally, regenerative medicine, such as stem cell therapy or tissue engineering, could help restore native heart function, thereby reducing mechanical support dependence. Addressing these research gaps may lead to more effective individualized management for CS, enhancing patient outcomes and quality of life.

## Conclusions

In conclusion, MCS represents a critical advancement in the management of CS, offering life-saving interventions for patients who do not respond adequately to conventional therapies. This narrative review highlights the evolving landscape of MCS technologies, from IABPs to more advanced devices such as VADs and ECMO. The review underscores the importance of timely and appropriate use of these devices, tailored to individual patient needs, to improve outcomes and reduce mortality. Despite the progress, further research is essential to address existing gaps, optimize device selection and management, and further enhance patient outcomes. Continued innovation and a nuanced understanding of MCS can potentially transform the future of CS treatment, providing new hope for patients facing this challenging condition.
